# Fluorescent Carbon Dots: Fantastic Electroluminescent Materials for Light‐Emitting Diodes

**DOI:** 10.1002/advs.202001977

**Published:** 2021-02-10

**Authors:** Biao Zhao, Zhan'ao Tan

**Affiliations:** ^1^ Beijing Advanced Innovation Center for Soft Matter Science and Engineering State Key Laboratory of Organic‐Inorganic Composites Beijing University of Chemical Technology Beijing 100029 China

**Keywords:** carbon dots, electroluminescence, fluorescence, light‐emitting diodes

## Abstract

Fluorescent carbon dots (CDs) have emerged as fantastic luminescent nanomaterials with significant potentials on account of their unique photoluminescence properties, high stability, and low toxicity. The application of CDs in electroluminescent light‐emitting diodes (LEDs) have aroused much interest in recent years. Herein, the state‐of‐the‐art advances of CD‐based electroluminescent LEDs are summarized, in which CDs act as active emission layer and interface transport layer materials is discussed and highlighted. Besides, the device structure of CD‐based LEDs and preparation methods of CDs are also introduced. Furthermore, the opportunities and challenges for achieving high performance CD‐based electroluminescent LED devices are presented. This review article is expected to stimulate more unprecedented achievements derived from CDs and CD‐based electroluminescent LEDs, thus further promoting their practical applications in future solid‐state lighting and flat‐panel displays.

## Graphical abstract

The application of fluorescent carbon dots (CDs) in electroluminescent light‐emitting diodes (LEDs) have attracted tremendous attention within the last few years. Recent advances concerning CD‐based LEDs are systematically summarized, with an emphasis on the employment of CDs as active emission layer and interface transport layer. Additionally, opportunities and challenges for future achieving high performance CD‐based electroluminescent LED devices are discussed.

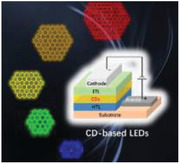

## 1 Introduction

Light‐emitting diodes (LEDs), now widely used in consumer electronics, have been regarded as evolutionary innovation in the fields of lighting and displaying due to their higher current‐to‐light efficiency, improved contrast, and larger color space than conventional liquid‐crystal displays. Recently, quantum dots and perovskite nanocrystals based LEDs have aroused extensive attention of the worldwide researchers and great achievements focusing on high‐performance devices of this kind have been achieved in the past few years.^[^ ^1^, ^2^, ^3^, ^4^, ^5^, ^6^, ^7^, ^8^ ^]^ However, the high‐toxicity, low‐stability, and high‐cost problems in the heavy‐metal (such as Cd^2+^ and Pb^2+^) based LEDs cannot be circumvented. These tough problems may seriously hamper their further developments and practical uses in consumer markets. Therefore, with the increasing attention to the environmental safety and human health, developing new luminescent materials with non‐toxic and good stability and employing them in LED devices is urgently needed.

Fluorescent carbon dots (CDs) are typical 0D nanocarbons with sizes usually less than 10 nm. Generally, CDs are free of heavy metals and constituted of abundant organic elements like carbon, hydrogen, nitrogen, and oxygen.^[^ ^9^ ^]^ As a typical member of carbon family, CDs own unique color‐tunable photoluminescence (PL) emission due to the size effect, surface effect, and edge effect,^[^ ^9^ ^]^ of which is rarely observed in graphene, fullerene, and carbon nanotube. The first discovery of CDs can be traced back to 2004, Xu et al. obtained fluorescent carbon during the purification of single‐walled carbon nanotubes.^[^ ^10^ ^]^ In 2006,Sun et al. achieved luminescent carbon nanoparticles via laser ablation of carbon target and first named them as “carbon dots.”^[^ ^11^ ^]^ Since then, massive efforts have been put to the investigations of CDs and various high‐quality CDs with color‐tunable PL emission from deep ultraviolet to near‐infrared have been reported successively.^[^ ^12^, ^13^, ^14^, ^15^, ^16^, ^17^ ^]^ More interestingly, CDs with unique room temperature phosphorescence (RTP)^[^ ^18^, ^19^, ^20^, ^21^, ^22^, ^23^ ^]^ and thermally activated delayed fluorescence (TADF)^[^ ^24^, ^25^, ^26^ ^]^ behaviors have also been discovered. Particularly, compared with organic fluorescent compounds, CDs own the merits of low toxicity, high stability, low cost, abundant raw materials and good solution‐processability.^[^ ^27^, ^28^, ^29^, ^30^ ^]^ These outstanding features endow CDs as excellent fluorescent nanomaterials with significant applications in bio‐imaging, theranostics, fluorescent sensing, photoelectric devices, and so on.^[^ ^31^, ^32^, ^33^, ^34^, ^35^, ^36^, ^37^, ^38^, ^39^ ^]^

With the rapid development of high‐quality CDs in recent years, studies on CD‐based LEDs have drawn increasing attention and a growing number of researchers have focused on this fast‐growing research field.^[^ ^40^ ^]^ Nowadays, CDs‐based LEDs have been prepared by two general strategies: CDs as phosphors and CDs as active emitters. The former is realized by using CDs as phosphors on a GaN‐based UV or blue LED chip, which is the most widely used method to achieve CDs‐based multicolor and white LEDs so far.^[^ ^41^, ^42^, ^43^, ^44^, ^45^ ^]^ Especially, thanks to the unremitting efforts by numerous researchers, the optimal device performance of phosphor‐based LEDs has already reached the standard of practical application. However, this approach highly relies on the intrinsic quality of the LED chips. Besides, this kind of LEDs merely utilize the photoluminescence property of CDs. Actually, employing CDs as emissive layer in electroluminescent LEDs is the most promising application for flat‐panel displays.

In view of the great achievements concerning CD‐based electroluminescent LEDs in the past years, a review article is thus highly necessary at present, which is expected to activate and promote further progress in this unique research area. To the best of our knowledge, though some review articles concerning the synthesis, physicochemical properties, surface functionalization, and applications of CDs have already been reported in literatures,^[^ ^9^, ^46^, ^47^, ^48^, ^49^, ^50^, ^51^, ^52^, ^53^, ^54^, ^55^, ^56^, ^57^, ^58^, ^59^, ^60^, ^61^, ^62^ ^]^ there is no report primarily focusing on the CD‐based electroluminescent LEDs. In the present article, we systematically summarize the recent advances of CD‐based electroluminescent LEDs, in which CDs act as emission layer and transport layer materials in LEDs is discussed and highlighted. Besides, the CD‐based LED device structure and preparation methods of CDs are briefly introduced. Furthermore, the opportunities and challenges for achieving high performance CD‐based electroluminescent LED devices are also presented and discussed. We believe that this review will not only be helpful in the development of CD‐based electroluminescent LEDs but will also be beneficial for the further development of organic electronics.

## 2 The Basic Concepts of CD‐Based Electroluminescent LEDs

**Figure 1:**
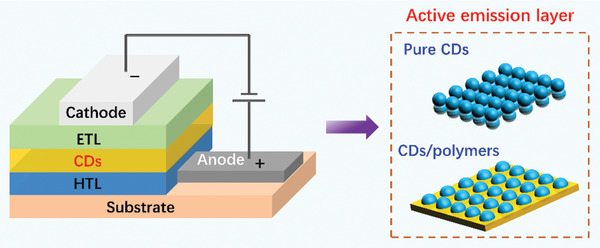
Illustrations of the typical device structure of CD‐based electroluminescent LEDs.

CD‐based electroluminescent LEDs possess similar sandwich device structure as quantum dot‐based LEDs (QLEDs), perovskite‐based LEDs (PeLEDs) and organic LEDs (OLEDs), in which CDs serve as middle emission layer surround by interface transport layers and electrodes. In a typical device structure of CD‐based electroluminescent LEDs, it can be divided into five parts: namely, an anode, a hole transport layer (HTL), an active emission layer, an electron transport layer (ETL) and a cathode, as illustrated in **Figure** **1**. Generally, the active emission layer can be constructed by pure CDs or CDs/polymers host‐guest complexes. The electrodes and part of transport layer materials are processed by thermal evaporation, while the CD‐based active emission layers and some organic conjugate buffer layer materials are processed by solution processing. Hence, the solubility of CDs in solvents is of significant importance in constructing LED devices. During the operation of LEDs, holes and electrons are first transfused into the ETL and HTL, respectively, and then recombined at the active emission layer to trigger light emission. By changing the emission wavelength of CDs, multicolor LED devices can be conveniently achieved.

For electroluminescent LEDs, electroluminescence (EL) spectra, luminance, turn‐on voltage, luminescent efficiency, and lifetime are critical parameters for evaluating the device performance. The EL spectra are used to provide the actual emission information (emission color and color chromaticity) of LEDs, in which different EL wavelengths are related to different emission color. Color chromaticity is a widely used parameter for representing the color purity of LEDs, which can be quantitatively calculated by Commission Internationle de L'Eclairage (CIE) 1931 color coordinates. Luminance is the unit area intensity of luminous perpendicular to the direction of beam propagation. Turn‐on voltage is the minimum voltage required to drive the current across the diode and start light‐emitting. Lifetime is a key index restricting the practical application of LED devices, which is defined as the time for the device performance to fall by 50% and can be measured by the *T*_50_ metric. Luminescent efficiency is a parameter to evaluate the photoelectric conversion efficiency of LEDs devices. Generally, external quantum efficiency (EQE), the ratio of the emitted photons from the device to the injected electrons, is used to weight the luminescent efficiency. Constructing electroluminescent LEDs with narrow‐bandwidth emission, low turn‐on voltage, high luminance, long lifetime, and high EQE is of great significance in practical applications.

## 3 Preparation Methods of CDs Used in Electroluminescent LEDs

To date, there are two typical strategies for preparing fluorescence CDs; that is, top‐down and bottom‐up methods. Generally, top‐down method is conducted by splitting large carbon species into fine ones through physical or electrochemical means. The carbon sources can be carbon nanotubes,^[^ ^63^ ^]^ graphite,^[^ ^11^ ^]^ carbon soot,^[^ ^64^ ^]^ activated carbon,^[^ ^65^ ^]^ graphite oxide,^[^ ^66^ ^]^ et al. However, these approaches are invariably tedious, uncontrollable, and expensive. Meanwhile, the resulting CDs generally possess low quantum yields (QYs). Nowadays, bottom‐up route is the most popular strategy for preparing CDs. It can be conducted through carbonization treatment of small organic molecules. By choosing cheap and well‐structured precursors such as citric acid, urea and phenylenediamine,^[^ ^12^, ^13^, ^14^ ^]^ various high‐quality CDs with multicolor emission and high QYs have been realized consecutively. Moreover, the obtained CDs are composed of amorphous to crystalline cores with plenty of functional groups like hydroxyl, amino, carboxyl, carbonyl, and epoxy on their surface.

**Figure 2:**
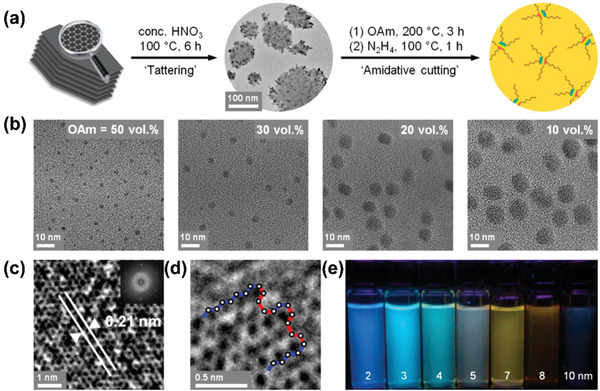
a) Schematic drawing for preparing GQDs by tattering and amidative cutting steps. b) TEM images of the GQDs. High‐resolution TEM images showing c) lattice spacing and d) edge structure of the GQDs. e) Fluorescence photographs of the GQDs under UV‐365 light. Reproduced with permission.^[^ ^76^ ^]^ Copyright 2014, American Chemical Society.

**Table** **1** summarizes the CDs used in electroluminescent LEDs. Based on the different preparation routes, the resulting CDs can be also named as graphene quantum dots (GQDs) and carbon quantum dots (CQDs). Generally, GQDs are comprised of regular hexagonal lattice of sp^2^ carbon cores and functional groups (such as hydroxy and carboxyl) on their edges.^[^ ^67^, ^68^, ^69^, ^70^, ^71^ ^]^ Compared with 2‐D graphene sheets such as graphene oxide, GQDs own unique fluorescence emission due to quantum confinement^[^ ^72^, ^73^ ^]^ and edge effects.^[^ ^74^, ^75^ ^]^ Kwon et al. used two‐steps method to synthesize GQDs with controllable size distributions.^[^ ^76^ ^]^ As shown in **Figure** **2**, tattered graphite sheets were first obtained by mildly oxidizing graphite with nitric acid (tattering step). Subsequently, the tattered graphite flakes were subject to oleylamine in an organic system and then underwent hydrazine treatment to eliminate superfluous oxygen‐containing carbons (amidative cutting step). By adjusting the amine concentration, the GQDs’ size could be easily tuned in the range of 2–10 nm. Moreover, with the increase of GQDs’ size, the energy gaps were gradually reduced, leading to the emission color varying from blue, cyan to brown.

**Table 1:** Overview of the reported CDs in electroluminescent LEDs

Product type	Carbon Source	Synthesis Method	Size	Emission Peak [nm]	QY [%]^a)^	Ref.
GQDs	Graphene sheets	Hydrothermal	5–15	510	–	^[^ ^71^ ^]^
GQDs	Graphite	Solvothermal	1–5	430–490	4	^[^ ^85^ ^]^
GQDs	Graphite	Amidative cutting	2–10	450	10	^[^ ^76^ ^]^
GQDs	Graphene oxide sheets	Hydrothermal	≈7	500	–	^[^ ^86^ ^]^
GQDs	Graphite	Microwave‐assisted hydrothermal	2.5	445	–	^[^ ^83^ ^]^
GQD	Graphite oxide	Amidative cutting	≈3	510, 570, 620	–	^[^ ^17^ ^]^
CQDs	Citric acid and diaminonaphthalene	Solvothermal	1.9–6.7	430–604	12–75	^[^ ^80^ ^]^
CQDs	Phloroglucinol	Solvothermal	1.9–3.9	472–498	54–72	^[^ ^77^ ^]^
CQDs	Banana leaves	Hydrothermal	4–6	425	–	^[^ ^95^ ^]^
CQDs	*N,N*‐dimethyl (diethyl and dipropyl)‐p‐phenylenediamine	Solvothermal	≈2.2	637, 642, 645	77.9, 85.2, 86.0	^[^ ^89^ ^]^
CDs	Citric acid and 1‐hexadecylamine	Pyrolysis	2–8	420–474	40–60	^[^ ^78^, ^79^, ^81^, ^87^ ^]^
CDs	Citric acid and diaminonaphthalene	Solvothermal and amination	2.4	433	70±10	^[^ ^93^ ^]^

^a)^ “–” means the corresponding QYs are not reported in the publications.

**Figure 3:**
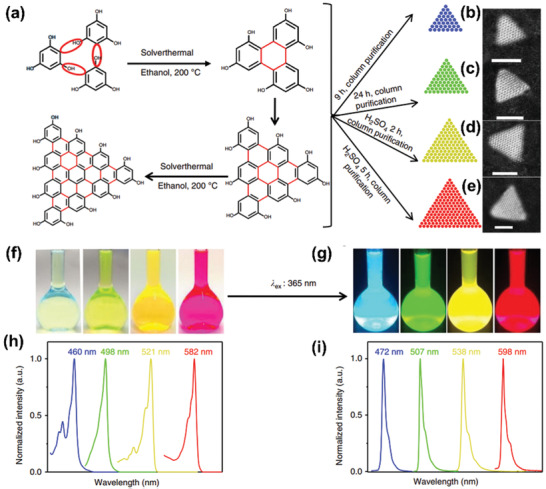
a) Synthesis route and b–e) the typical aberration‐corrected HAADF‐STEM images of the narrow bandwidth emission triangular CQDs. Photographs of the triangular CQDs solution under f) daylight and g) UV‐365 light. h) The normalized UV–vis absorption and i) PL spectra of the multicolored triangular CQDs. Reproduced with permission.^[^ ^77^ ^]^ Copyright 2018, Springer Nature.

CQDs are generally constructed by bottom‐up routes and possess obvious crystal lattices corresponding to the interplanar spacing of graphene. Usually, CQDs show intrinsic state luminescence (excitation wavelength‐independent fluorescence) and quantum confinement effect (size‐dependent emission), in which PL emission comes from conjugated *π*‐domains.^[^ ^9^ ^]^ Recently, Yuan et al. achieved high‐quality LEDs based on multicolored triangular CQDs with narrow full width at half maximum (FWHM) of 29–30 nm and high QYs (54–72%).^[^ ^77^ ^]^ The triangular CQDs were prepared by a bottom‐up solvothermal treatment using phloroglucinol as precursor (**Figure** **3**). Due to the quantum confinement effect, the emission color changed from blue, green, yellow to red with the increase of CDs’ sizes from 1.9 to 3.9 nm, along with the optical bandgap energy decreased from 2.63 to 2.07 eV. Besides, density functional theory (DFT) calculations were used to investigate the structural information of the triangular CQDs. The obtained results demonstrated that the high color‐purity PL emission was attributed to the surfaces’ hydroxyl groups, through effectively enhancing the charge delocalization and simultaneously restricting the photons and electrons coupling. Actually, the physicochemical properties of the resulting CDs are extremely depended on the choice of precursor kinds and preparation methods.

Despite different preparation methods and precursors may lead to CDs with different varieties and diverse structures, the optical properties of CDs are usually similar. There are many excellent review papers provide comprehensive overviews on the structure, optical properties, and applications of CDs,^[^ ^9^, ^46^, ^47^, ^48^, ^49^, ^50^, ^51^, ^52^, ^53^, ^54^, ^55^, ^56^, ^57^, ^58^, ^59^, ^60^, ^61^, ^62^ ^]^ thus the optical properties of CDs are briefly introduced in this contribution. Generally, CDs show strong light absorption in the UV–vis region due to the presence of conjugated sp^2^ atomic core and numerous functional groups or polymer chains at the edge. Especially, the absorption wavelength can be altered by changing the CDs’ size, doping, and surface modification, accompanied by the change of fluorescence emission. Actually, the most valuable merit of CDs is the unique color‐tunable photoluminescence emission. Thanks to the unremitting efforts on preparing fluorescent CDs through selecting different precursors, optimizing reaction, conditions, and post‐modification, great achievements concerning high‐quality CDs have been achieved in the past years. Nowadays, multicolor CDs with both high quantum yields and tunable PL emission from deep ultraviolet to near‐infrared have been reported successively.^[^ ^12^, ^13^, ^14^, ^15^, ^16^, ^17^ ^]^ Nevertheless, owing to the complex composition and multifarious structure of CDs, it remains a big challenge to reveal the exact luminescent mechanisms of CDs. To date, four popular luminescence mechanisms of CDs have been widely accepted: bandgap transitions from conjugated *π*‐domains, surface‐state emission, molecular‐state emission, and crosslink enhanced emission effect.^[^ ^59^ ^]^ Owing to the different photoluminescence mechanisms, CDs usually show different emission behavior; that is, excitation wavelength‐independent luminescence and excitation wavelength‐dependent luminescence. Besides, apart from fluorescence performance, rapidly growing interest has been devoted to studies dealing with CDs with room temperature phosphorescence and thermally activated delayed fluorescence, which will be discussed in Section 5.

## 4 Recent Advances in CD‐Based Electroluminescent LEDs

In the past decade, the application of CDs in electroluminescent LEDs has been extensively investigated. The related device structure and device performance are summarized in **Table** **2**. Currently, there are three main routes for employing CDs in electroluminescent LEDs, including pure CDs serving as emission layer, doped CDs serving as emission layer, and CDs serving as interface transport layer. The detail advances of CD‐based electroluminescent LEDs are introduced below.

**Table 2:** Overview of the device performance of CD‐based electroluminescent LEDs

Year	Device structure	Color	*V*_ON_ (V)^a)^	*L*_max_ (cd m^−2^)^a)^	*η*_c_ (cd A^−1^)^a)^	EQE (%)^a)^	Ref.
2011	ITO/PEDOT:PSS/CDs/TPBI/LiF/Al	White	6	35	0.022	0.083	^[^ ^78^ ^]^
2013	ITO/PEDOT:PSS/Poly‐TPD/CDs/TPBI/LiF/Al	Blue	–	24	0.03	–	^[^ ^81^ ^]^
		White	–	90	0.035	–	
2014	ITO/PEDOT:PSS/PVK:GQDs/TPBI/LiF/Al	Blue	8	>1000	0.65	–	^[^ ^85^ ^]^
2014	ITO/GraHIL/CBP:GQDs/TPBI/LiF/Al	White	–	–	–	0.1	^[^ ^76^ ^]^
2015	ITO/PEDOT:PSS/PVK:GQDs/LiF/Al	White	–	1	–	–	^[^ ^86^ ^]^
2016	ITO/PEDOT:PSS/CBP:GQDs/TPBI/LiF/Al	White	5	200	–	0.24	^[^ ^83^ ^]^
2016	ITO/SoHIL/TCTA:TPBI:GQDs/TPBI/Al	Green	–	390	3.47	1.28	^[^ ^17^ ^]^
		Yellow	–	3	–	0.1	
		Red	–	2	–	0.1	
2016	ITO/PEDOT:PSS/CQDs/TPBI/Ca/Al	Blue	4.7	136	0.084	–	^[^ ^80^ ^]^
		Green	4.5	93	0.045	–	
		Yellow	4.2	60	0.02	–	
		Orange	3.9	65	0.027	–	
		Red	3.7	12	0.0028	–	
2016	ITO/PEDOT:PSS/PVK:CQDs/TPBI/Ca/Al	White	3.9	2050	1.1	–	^[^ ^80^ ^]^
2018	ITO/PVK:CDs/TPBI/LiF/Al	Blue	8.5	569.8	–	–	^[^ ^87^ ^]^
2018	ITO/PVK:CDs/TPBI or TmPyPB/LiF/Al	Yellow	–	339.5	–	–	^[^ ^88^ ^]^
		White	7	455.2	–	–	
2018	ITO/PEDOT:PSS/PVK:CQDs/TPBI/Ca/Al	Blue	4.7	1882	1.22	–	^[^ ^77^ ^]^
		Green	3.7	4762	5.11	–	
		Yellow	3.5	2784	2.31	–	
		Red	3.1	2344	1.73	–	
2019	ITO/ZnO/PEIE/CDs/PVK/MoO_3_/Au	White	5	27	0.06	–	^[^ ^79^ ^]^
2019	ITO/PEDOT:PSS/Poly‐TPD/PVK:CQDs/TPBI/Ca/Al	White	3	5909	3.85	–	^[^ ^89^ ^]^
		Red	3.6	2960	2.19	–	
2019	ITO/PEDOT:PSS/PVK:CQDs/TPBI/Ca/Al	Deep Blue	4.8	5240	2.6	4	^[^ ^93^ ^]^
2019	ITO/CQDs/CsPbBr_3_/Au	Green	2.8	25 770	41.1	13.8	^[^ ^94^ ^]^

^a)^ “–” means the corresponding device parameters are not reported in the publications.

### 4.1 Pure CDs Serving as Active Emission Layer

**Figure 4:**
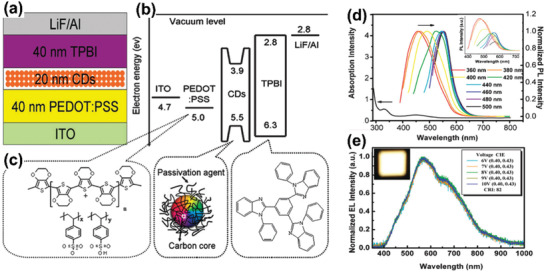
a) Schematic diagrams of the WLEDs’ cross‐section. b) The suggested energy band diagram of the WLEDs. c) Molecular structures of PEDOT:PSS and TPBI, and the schematic drawing of the CDs. d) The absorption and normalized PL spectra of the CDs thin film spin‐coated on silica glass. e) Normalized EL spectra of WLEDs at applied bias voltages. Reproduced with permission.^[^ ^78^ ^]^ Copyright 2011, Royal Society of Chemistry.

In 2011, Ma and coworkers reported the first electroluminescent device by using 1‐hexadecylamine passivated CDs as active emission layer (**Figure** **4**).^[^ ^78^ ^]^ To prepare LED devices, a HTL material, poly(3,4‐ethylenedioxythiophene):poly(styrenesulfonate) (PEDOT:PSS), was first spun‐casted onto the ITO glass. The purpose of this step was to increase the anode work function of ITO and simultaneously decrease its surface roughness. Then, the emissive layer of CDs was spun‐casted onto the PEDOT:PSS. Afterward, an ETL of 1,3,5‐tris(*N*‐phenylbenzimidazol‐2‐yl) benzene (TPBI) was deposited on the emission layer, followed by thermal evaporation of LiF and Al electrodes. Through optimizing the device structure, the authors obtained white‐color light‐emitting diodes (WLEDs) with a CIE coordinate of (0.40, 0.43) and color‐rendering index (CRI) of 82. The turn‐on voltage (*V*_on_) of the prepared WLEDs were found to be 6 V. Besides, under a current density of 5 mA cm^−2^, the WLEDs possessed a maximum EQE of 0.083%., which may be disadvantageous to practical application. The high *V*_on_ and low EQE was probably due to the presence of insulated 1‐hexadecylamine chains on the surface of CDs, which was unfavorable for the injection of electrons and holes. Besides, maximum luminance (*L*_max_) of 35 cd m^−2^ was obtained at 9 V, with a current efficiency (*η*_c_) of 0.022 cd A^−1^. Recently, using the same CDs as single emissive layer, Paulo‐Mirasol et al. realized solution processed inverted white‐color LEDs.^[^ ^79^ ^]^ The devices were constructed by using ZnO nanoparticles as ETL and poly(*N*‐vinylcarbazole) (PVK) as HTL.

**Figure 5:**
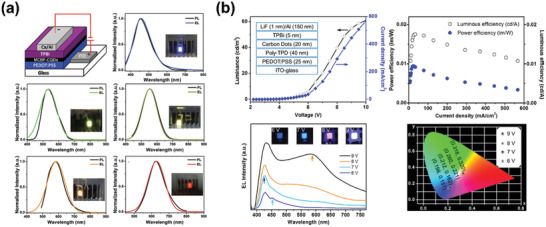
a) The device structure, normalized PL spectra, and the corresponding EL spectra of monochrome LEDs with blue, green, yellow, orange, and red emission. Reproduced with permission.^[^ ^80^ ^]^ Copyright 2016, Wiley‐VCH. b) The device performance including current density, brightness, luminous, and power efficiencies versus current density, EL spectra, true color photographs, and CIE 1931 coordinates of the CD‐based LEDs emitting blue, cyan, magenta, and white light. Reproduced with permission.^[^ ^81^ ^]^ Copyright 2013, American Chemical Society.

Achieving high‐quality bandgap fluorescent CDs is an effective method for realizing high performance electroluminescent LEDs. Taking citric acid and diaminonaphthalene as precursors, our group has succeeded in preparing multi‐color bandgap fluorescent CQDs (MCBF‐CQDs) though a solvothermal reaction.^[^ ^80^ ^]^ The MCBF‐CQDs showed bright PL emission from blue to red. By taking the MCBF‐CQDs as active emission layer materials, we first realized monochrome LEDs with blue, green, yellow, and red color, under a typical device structure of ITO/PEDOT:PSS/MCBF‐CQDs/TPBI/Ca/Al (**Figure** **5**a). Thanks to the outstanding bandgap emission, the obtained LEDs all exhibited stable and voltage‐independent EL emission. Among the multicolor LEDs, the blue LEDs exhibited the best device performance, with *η*_c_ of 0.084 cd A^−1^ and *L*_max_ of 136 cd m^−2^. Besides, with the color varying from blue to red, the *V*_on_ of LEDs reduced from 4.7 to 3.7 V, due to the decreased hole injection energy barrier between MCBF‐CQDs and PEDOT:PSS.

Expect for achieving colorful LEDs based on multicolor CDs, switchable EL behavior, and white emission can be also obtained in monochrome CDs emissive layer structured LEDs. Zhang and colleagues prepared a series of color‐switchable LEDs from CDs of the same size via adjusting the injected current density and device structure.^[^ ^81^ ^]^ They found that when increasing the applied voltage from 6 V to 9 V, the LED exhibited three emission peaks at 426, 452, and 588 nm respectively, leading to the emission color of LEDs changed from blue, cyan, magenta to white (Figure 5b). For blue and white color LEDs, the maximum brightness was 24 and 90 cd m^−2^ respectively. Time‐resolved photoluminescence results demonstrated that the observed color‐switchable emission behavior was probably due to the multiple‐recombination process. This study provides a new idea for achieving polychromatic displays from single pixel CDs.

### 4.2 Doped CDs Serving as Active Emission Layer

According to the above investigations we can find that the maximum brightness of CD‐based electroluminescent LEDs with pure CDs as emissive layer is lower than 150 cd m^−2^, which is far from our expectation and unrealistic in practical applications. The main reason for this result is due to the aggregation‐induced quenching (AIQ) effect of CDs.^[^ ^82^ ^]^ In most situations, CDs will undergo significant fluorescence quenching in solid state because of excessive resonance energy transfer and strong *π*–*π*‐interactions. As a result, the device performance of CD‐based LEDs inevitably reduces. Doping CDs in host materials may be an effective strategy to prevent the AIQ effect and simultaneously reduce the roughness of the emission film, thus providing an effective access to realize high‐quality CD‐based electroluminescent LEDs.

**Figure 6:**
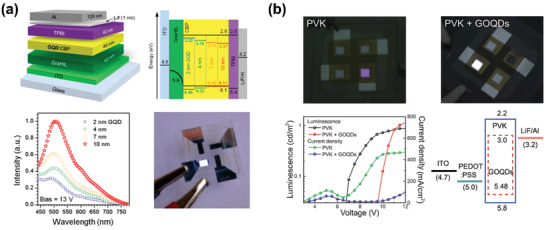
a) Physical and electronic structures, normalized EL intensity and photo of the GQDs‐based LEDs. Reproduced with permission.^[^ ^76^ ^]^ Copyright 2014, American Chemical Society. b) Digital camera images, current density and luminance versus voltage curves and schematic energy diagram of LEDs using pristine PVK and GQDs blended PVK as emissive layers, respectively. Reproduced with permission.^[^ ^86^ ^]^ Copyright 2015, Springer Nature.

Taking advantage of the doping strategy, Rhee and coworkers realized LED device based on GQDs.^[^ ^76^ ^]^ A series of GQDs with size distributions from 2 nm to over 10 nm were prepared through amidative cutting of graphite. The authors found the PL emission of such GQDs has a close relationship with its energy gaps. Detailedly, the PL color changed from blue to brown with the narrow down of energy gaps. By doping the prepared GQDs in a host material of 4,4′‐bis(carbazol‐9‐yl)biphenyl (CBP), electroluminescent devices with a maximum EQE of ≈ 0.1% were fabricated (**Figure** **6**a). The poor device performance was probably due to the low QYs (≈10%), deep highest occupied molecular orbital (HOMO) levels of the as‐prepared GQDs and the presence of bulky ligand molecules, which were disadvantageous for the efficient charge injection and energy transfer process. Similarly, taking CBP as host, Luo et al. reported solution‐processed WLEDs using white fluorescent GQDs doped CBP as the emissive layer.^[^ ^83^ ^]^ When the doping concentration of GQDs was 10 wt%, best device parameters were achieved, with an EQE_max_ of 0.2 % and a maximum luminance of 200 cd m^−2^. In the above systems, electrons and holes were first injected into CBP and then transferred from the host CBP to the guest GQDs. It implies that the energy transfer process between the host materials and the emissive GQDs is of great importance for achieving bright electroluminescence. Therefore, choosing proper host materials is also very important for obtaining high‐quality LED devices.

Due to the high stability, good filming property and proper energy levels, PVK is widely used as hole transport material in photodevices. The HOMO and lowest unoccupied molecular orbital (LUMO) energy levels of PVK are −2.2 and −5.8 eV, respectively.^[^ ^84^ ^]^ Taking PVK as host material, Jeon's group prepared GQD‐based electroluminescent LEDs using a device structure of ITO/PEDOT:PSS/PVK:GQDs/TPBi/LiF/Al.^[^ ^85^ ^]^ Under 3.0 wt% GQDs doping, high‐performance LEDs with a luminance over 1000 cd m^−2^ and luminous efficiency of 0.65 cd A^−1^ were obtained. It was found the enhancement of device performance was due to presence of PVK, which could provide additional carrier transport/injection channels and simultaneously increase the odds of radiative recombination from GQDs.

To more clearly understand the light‐emitting mechanism, Park and coworkers prepared LED devices with a simple structure of ITO/PEDOT:PSS/PVK:GQDs//LiF/Al (Figure 6b).^[^ ^86^ ^]^ By using this simple device structure, the influence from other function layers on the electroluminescence could be eliminated, and thereby the origin of electroluminescence could be revealed. The authors found that the investigated GQDs exhibited green PL emission at 500 nm in solution state, but white emission was observed in LED device. Laboratory tests together with computer simulations were carried out to investigate the electroluminescence process. Experiment results demonstrated that the white EL emission was caused by the formation of hybridized GQD‐PVK complex emission. Based on the results, Park and coworkers proposed that hybridization complex states can be formed between carbazole‐based host materials and GQDs, which is beneficial for generating white emission. This work provides an alternative pathway for preparing WLEDs. Following this idea, more WLEDs can be easily achieved. However, because of the absence of transport layer materials, the LEDs performance prepared in the simple structure was relatively poor, with a maximum luminance of only 1 cd m^−2^.

**Figure 7:**
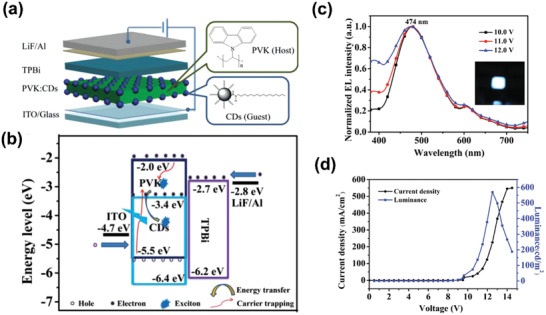
a) A schematic illustration of CD‐based LEDs’ structure. b) The energy level structure and emission mechanism of CD‐based LEDs. c) The normalized EL spectra and d) the current density–voltage–luminance characteristics curve of the CD‐based LEDs. Reproduced with permission.^[^ ^87^ ^]^ Copyright 2018, Wiley‐VCH.

Following the host‐guest energy transfer emission mechanism, Yang, Liu, and coworkers realized blue CD‐based electroluminescent LEDs using PVK as host and oleophylic CDs as dopant,^[^ ^87^ ^]^ as illustrated in **Figure** **7**. The CDs were prepared through microwave carbonization means, with 1‐hexadecylamine as passivant and citric acid as carbon source. They found the doping concentration played an important effect on the EL spectra of LEDs. Specifically, the EL emission from PVK decreased gradually with the increase of doping concentration, accompanied with the increase of EL emission from blue CDs. By optimizing the CDs concentration, a maximum luminance of 569.8 cd m^−2^ was obtained. Later, using the same blue‐CDs as emissive layer, the same group fabricated yellow‐color and white‐color CD‐based electroluminescent LEDs by tactfully altering the device structure and tuning the doping concentration of CDs.^[^ ^88^ ^]^ The prepared WLEDs exhibited a maximum luminance of 455.2 cd m^−2^, together with CIE coordinates of (0.29, 0.33) and high CRI of 83. Besides, maximum luminance of 339.5 cd m^−2^ was obtained in the yellow‐color LEDs. Regretfully, the prepared LEDs all showed relatively high turn‐on voltages. The cause of this phenomenon was probably due the presence of long passivating chains on the surface of CDs, which is adverse for the balance of electron and hole injections. Therefore, during the fabrication of CD‐based electroluminescent LEDs, the surface property of CDs themselves should be also taken into account.

**Figure 8:**
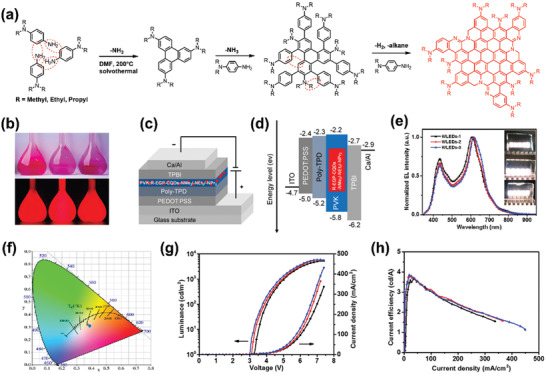
a) Synthesis and b) photographs of R‐EGP‐CQDs. c) The device structure and d) energy level diagram of WLEDs. e) EL spectra, operation photographs and f) corresponding CIE coordinates of WLEDs. (g) The current density–voltage–luminance and h) current efficiency of WLEDs. Reproduced with permission.^[^ ^89^ ^]^ Copyright 2019, Wiley‐VCH.

Generally, efficient carrier injection is crucial for the construction of electroluminescent LEDs. However, in most cases, applying excitation‐dependent fluorescence CDs as emissive layer is disadvantageous for the carrier injection, primarily due to the molecule‐state and surface‐defect‐state fluorescence emission mechanisms.^[^ ^69^, ^71^ ^]^ Fabricating high‐quality bandgap fluorescent CDs with excitation‐independent fluorescence is a promising strategy for constructing high‐quality CD‐based electroluminescent LEDs. Using green‐color CDs blended PVK as active emission layer, our group achieved WLEDs with a CIE color coordinate of (0.30, 0.33) and low turn‐on voltage of 3.9 V.^[^ ^80^ ^]^ Moreover, the *η*_c_ and *L*_max_ of the prepared WLEDs could reach up to 1.1 cd A^−1^ and 2050 cd m^−2^ respectively, which are comparable to semiconductor QLEDs. Recently, we achieved three red‐color electron‐donating group passivated CQDs (R‐EGP‐CQDs) with high QY up to 86.0% (**Figure** **8**).^[^ ^89^ ^]^ Based on the R‐EGP‐CQDs, solution‐processed electroluminescent warm‐WLEDs were obtained. Through optmizing the device structures, a maximum current efficiency of 3.85 cd A^−1^ and maximum luminance of 5909 cd m^−2^ were realized, which is the best device performances among the reported CDs‐based WLEDs. Besides, the warm‐WLEDs all possessed good long‐time operation stability.

Color purity is an important parameter in light displays, which is often specified by FWHM. In spite of the intensive studies on CDs, it has been generally considered that fluorescent CDs can only exhibit broad FWHM higher than 60 nm.^[^ ^12^, ^13^, ^14^, ^15^, ^16^, ^90^, ^91^ ^]^ It is reasonable that CDs can be regarded as mixtures of variable‐sized carbon dots, in which different size is corresponding to the different PL emission wavelength. Based on this consideration, obtaining CDs with uniform size distribution seems to be an effective pathway to realize narrow bandwidth emission. However, subsequent numerous investigations have proved that the FWHM still remains broad even after uniforming the CDs’ size through precise purification process. It means the broad FWHM may be caused by the intrinsic property of CDs rather than the size polydispersity. As we know, broad bandwidth emission is disadvantageous for the preparation of high color‐purity CD‐based electroluminescent LEDs.^[^ ^92^ ^]^ Thus, exploiting novel narrow bandwidth emission CDs is urgently needed.

**Figure 9:**
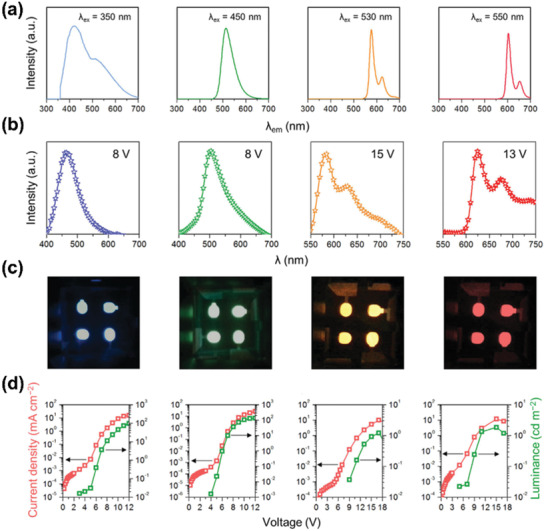
a) PL spectra of bare GQDs and functionalized GQDs. b) EL spectra, c) photographs and d) current density and luminance of the corresponding GQD‐based LEDs. Reproduced with permission.^[^ ^94^ ^]^ Copyright 2016, Springer Nature.

Despite the intense studies dealing with CDs in the last decade, investigations on narrow‐band emissive CDs are sporadically reported so far and there is no general rule to guide the preparation of narrow‐band emissive CDs due to lacking experimental samples and theoretical knowledge. According to the literatures, the choice of raw materials is crucial for preparing narrow‐band emissive CDs. Yang and coworkers reported deep red emissive CDs with a FWHM of 20 nm from taxus leaves.^[^ ^17^ ^]^ Besides, reaction solvents also play an important role on the FWHM. Xiong and coworkers prepared full‐color CDs with FWHM in the range of 55–108 nm by adjusting the reaction solvents.^[^ ^31^ ^]^ Lin and colleagues prepared near‐infrared emissive CDs with FWHM of ≈30 nm through microwave heating of glutathione.^[^ ^93^ ^]^ Particularly, they found the narrow‐band emissive CDs could be only constructed in formamide solvent. Furthermore, post‐modification is also an effective method to narrow the emission band of CDs. In view of the broad bandwidth emission of GQDs, which is due to the presence of abundant energy gaps and electronic states caused by residual oxygen and nitrogen groups, Kwon et al. found that chemical functionalization of GQDs using aniline derivatives could narrow the FWHM by producing new extrinsic energy levels.^[^ ^94^ ^]^ To confirm this point, they modified bare GQDs by taking 6‐aminoquinoline, 4‐(methylthio)aniline and 4‐methoxyaniline as modified agents. After functionalization, the GQDs exhibited narrow photoluminescence linewidths with FWHM in the range of 20–50 nm (**Figure** **9**). Utilizing the functionalized GQDs as emissive layers, Kwon et al. further fabricated green, orange, and red‐colored LEDs, with best *L*_max_ of 390 cd m^−2^, EQE_max_ of 1.28% and maximum current efficiency of 3.47 cd A^−1^ in green LEDs.

**Figure 10:**
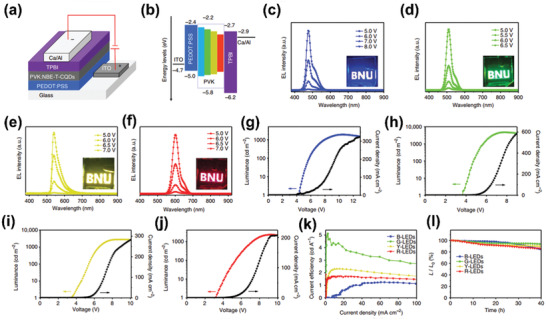
a) The device structure and b) energy level diagram of the NBE‐T‐CQDs‐based LEDs. EL spectra of the c) B‐, d) G‐, e) Y‐, and f) R‐LEDs at different bias voltage, respectively. The maximum luminance–current–voltage characteristic of g) B‐, h) G‐, i) Y‐, and j) R‐LEDs, respectively. k) The current efficiency versus current density and l) the stability plots of the B‐, G‐, Y‐, and R‐LEDs. Reproduced with permission.^[^ ^77^ ^]^ Copyright 2018, Springer Nature.

Recently, our group achieved high‐quality triangular CQDs (NBE‐TCQDs) by using phloroglucinol as precursor via solvothermal method.^[^ ^77^ ^]^ The obtained multicolored NBE‐TCQDs all possessed narrow FWHM of ≈30 nm, with high QYs in the range of 54–72%. To explore the origin of the high color‐purity and narrow bandwidth, specific characterizations focusing on the optical and structural properties of the NBE‐TCQDs together with theoretical calculations were carried out. The obtained results revealed that the high color‐purity was derived from the perfect triangular crystalline. Notably, CDs have always been thought to be spherical and ellipsoidal, it is the first report concerning on triangular CDs. This work undoubtedly opens a new door for the future development of CDs. Moreover, taking advantage of ITO/PEDOT:PSS/PVK:NBE‐TCQDs/TPBI/Ca/Al device structure, multicolored LEDs were further fabricated by taking the NBE‐TCQDs as emissive materials, with high current efficiency in the range of 1.22–5.11 cd A^−1^ and maximum luminance of 1882–4762 cd m^−2^ (**Figure** **10**). This study represents a critical step forward for high performance LEDs. Furthermore, by skillfully designing the molecule structure of precursors, other special‐shaped CDs with outstanding photophysical properties can be expected.

Besides, theoretical study can also assist the preparation of narrow‐band emissive CDs. Very recently, Yuan et al. established a two‐step strategy to fabricate narrow bandwidth deep‐blue emissive CDs by amination process.^[^ ^95^ ^]^ To get a better understanding of the broad emission mechanism of common CDs, the authors first investigated the influence of different functional groups (such as carboxyl and amino) on the emission bandwidth of CDs by using DFT calculations. DFT results revealed that large bandgap fluctuations are formed when carboxyl groups exist on the edge of CDs. However, if the carboxyl groups are replaced with amino groups, the bandgap fluctuations will reduce significantly. Generally, bandgap fluctuations are closely related to the magnitude of FWHM and larger bandgap fluctuations will lead to large FWHM. Based on the simulation results, the authors further passivated the broad emissive CDs using ammonia liquor and successfully obtained narrow‐bandwidth emissive CDs with PL emission at 433 nm. The resulting CDs exhibited narrow FWHM of 35 nm with a high QY up to 80% and color coordinate of (0.15, 0.05). Furthermore, taking advantage of the high color‐purity deep‐blue CDs, Yuan et al. achieved high performance LEDs with EQE_max_ of 4% and *L*_max_ of 5240 cd m^−2^. This study demonstrated that the presence of oxygen‐containing functional groups may cause spectral broadening due to the wave‐function polarization deriving from thermal vibrations and surface amination process may be an effective strategy for fabricating high color‐purity emissive CDs. Therefore, theoretical calculations together with experiment validation may be a powerful tool for future design and preparation of narrow‐band emissive CDs.

### 4.3 CDs Serving as Interface Transport Layer

**Figure 11:**
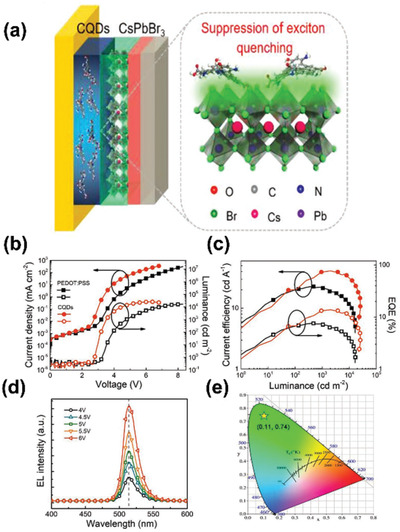
a) Schematic illustration of the possible passivation mechanism between CQDs and CsPbBr3. b) The current density–luminance–voltage, c) current efficiency and EQE curves of PeLEDs based on PEDOT:PSS HIL and CQDs HIL. d) EL spectra and e) CIE coordinates of PeLEDs based on CQDs HIL. Reproduced with permission.^[^ ^96^ ^]^ Copyright 2019, Wiley‐VCH.

Despite of serving as emissive layer, Wang et al. first demonstrated that CDs could serve as a new p‐type hole injection layer (HIL) in constructing LED devices.^[^ ^96^ ^]^ The applied CDs were prepared by solvothermal strategy using 2,3‐diaminonaphthalene and citric acid as starting materials. The HOMO, LUMO, and work function of the obtained CDs were calculated to be 5.6, 2.6 and 4.5 eV, respectively. Besides, the hole mobility of the CDs was ≈3.2 × 10^−4^ cm^2^ V^−1^ s^−1^, which was comparable to the conventional organic hole transport materials poly(9,9‐dioctylfluorene‐*co*‐*N*‐(4‐(3‐methylpropyl))diphenylamine) (TFB) and poly(*N,N*′‐bis(4‐butyl‐phenyl)‐*N,N*′‐bis(phenyl)‐benzidine) (poly‐TPD). The above results clearly demonstrated the good hole transport performance of the as‐prepared CDs. Subsequently, Wang et al. fabricated green CsPbBr_3_ PeLEDs by using the as‐prepared CDs as HIL to substitute PEDOT:PSS (**Figure** **11**). This process could promote the rapid hole injection from ITO glass to perovskite emission layer and thereby remarkably enhanced the charge balance. Besides, the authors found that the functional groups on the CDs’ surface could efficiently passivate the emissive perovskite and decrease the exciton quenching phenomenon at the PeLED interface. Benefited from the above advantages, green‐color PeLEDs with high performance and good stability were achieved. The maximum luminance, turn‐on voltage and EQE_max_ of the constructed PeLEDs were calculated to be 25 770 cd m^−2^, 2.8 V, and 13.8%, respectively. This work not only enhances our understanding on the electrooptical properties of CDs, but also provides more possibilities for applying CDs in optoelectronic devices.

Recently, Parmar and coworkers demonstrated that CDs could also be utilized as electron transport layer materials in fabricating LEDs.^[^ ^97^ ^]^ The CDs with size of ≈4–6 nm were prepared from banana leaves via one step hydrothermal process. To confirm the electron transport ability of CDs, the authors prepared two LEDs with device structure of ITO/PEDOT:PSS/PFO/LiF/Al and ITO/PEDOT:PSS/PFO/CD/LiF/Al, respectively. It was found that the introduction of CDs layer could obviously decrease the turn‐on voltages by facilitating the electron injection. Moreover, the LED device with CDs possessed higher stability and longer lifetime.

## 5 Research Opportunities for High Performance CD‐Based Electroluminescent LEDs

Compared with OLEDs, QLEDs, and PeLEDs, the device performance of reported CD‐based LEDs is far from satisfactory, with maximum luminance lower than 6000 cd m^−2^ and poor external quantum efficiencies (Table 2). Exploiting high performance CD‐based LEDs is urgently needed in consideration of the demands in practical applications. In view of the above content and our previous series of studies concerning CD‐based electroluminescent LEDs, we put forward several feasible strategies for constructing high performance CD‐based electroluminescent LEDs from the perspective of high‐quality CDs’ preparation and device structure optimization in this section. We expect the following introduction can offer more inspirations for scientists to develop novel CDs and further promote the development of CD‐based electroluminescent LEDs in the next decade.

### 5.1 Solid‐State Fluorescent CDs

In Section 4.1, we can find that pure CDs as emissive layer in LEDs generally leads to inferior device performance due to the AIQ effect. Though blending CDs into host materials can solve this question to some degree, the concentration quenching effect of CDs still inevitably exist. Moreover, the host‐guest method sometimes could result in the appearance of EL emission peak from host materials, leading to the broadening of EL spectra and poor color purity of LEDs. Therefore, to overcome this problem, it is necessary to exploit pure CDs with efficient solid‐state luminescence property, which will greatly promote their applications in solid‐state displays.

**Figure 12:**
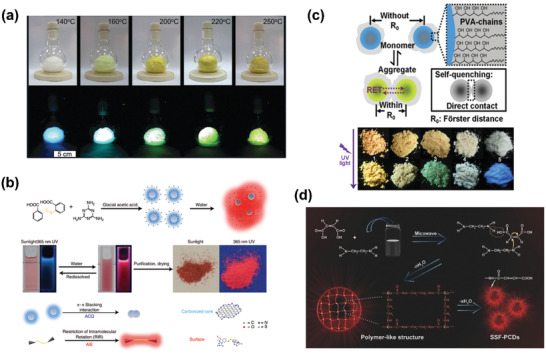
a) Photographs of the inflated solid‐state fluorescent CDs foams prepared by the vacuum heating method at different temperatures. Reproduced with permission.^[^ ^109^ ^]^ Copyright 2019, Royal Society of Chemistry. b) The schematic diagram of the preparation of hydrophobic carbon dots with blue dispersed emission and red aggregation‐induced emission. Reproduced with permission.^[^ ^110^ ^]^ Copyright 2019, Springer Nature. c) Schematic and photographs of self‐quenching‐resistance solid‐state CDs by one‐pot hydrothermal treatment of PVA and EDA. Reproduced with permission.^[^ ^82^ ^]^ Copyright 2015, Wiley‐VCH. d) The synthetic route of solid‐state fluorescent CDs from condensation crosslinking and carbonization of maleic acid and ethylenediamine. Reproduced with permission.^[^ ^111^ ^]^ Copyright 2017, Wiley‐VCH.

The key to obtain solid‐state fluorescent CDs is to overcome or prevent AIQ effect. In the past few years, massive endeavors have been made to achieve solid‐state fluorescent CDs toward developing suitable CDs‐based composites by using polymers,^[^ ^98^, ^99^, ^100^, ^101^ ^]^ starch,^[^ ^102^ ^]^ inorganic salt,^[^ ^91^, ^92^, ^93^, ^94^, ^95^, ^96^, ^97^, ^98^, ^99^, ^100^, ^101^, ^102^, ^103^, ^104^ ^]^ polyhedral oligomeric silsesquioxanes,^[^ ^105^ ^]^ and silica^[^ ^106^, ^107^ ^]^ as matrices. However, only CDs in low loading fractions can lead to high QY in these situations. Delightedly, Liu's group reported blue‐color B‐doped CDs by using boric acid and ethylenediamine as precursors through hydrothermal approach.^[^ ^108^ ^]^ It was found that the resulting B‐doped CDs could exhibit strong fluorescence emission in solid state, with a QY of 22%. Besides, Qu and coworkers developed a space‐confined vacuum heating route to prepare solid‐state fluorescent CDs in the presence of citric acid, urea and CaCl_2_ (**Figure** 12a).^[^ ^109^ ^]^ Upon controlling the heating temperature, green‐color CDs were obtained with QY up to 72% in solution. More excitingly, the obtained CDs could also exhibit strong fluorescence emission with a high QY of 65% in solid films. Recently, Yan et al. reported hydrophobic CDs with reversible two‐switch‐mode luminescence via solvothermal treatment of melamine, dithiosalicylic acid and acetic acid solution.^[^ ^110^ ^]^ The obtained CDs showed blue PL emission in disperse solutions but red aggregation‐induced emission behavior in solid power (Figure 12b). They found the blue emission was induced by the formation of hydrophobic CD clusters in solution, while red fluorescence would be turned on when *π*–*π* stacking interactions took place in the carbonized cores, primarily due to restriction of the surfaces’ intramolecular rotation around disulfide bonds. More interestingly, the blue and red emissions were dynamically reversible by simply turning the state (solution or solid) of CDs. This work provides a new pathway for applying CDs in dual encryption area.

Besides, introducing polymer chains into CDs can also lead to solid‐state fluorescent CDs. Lei, Liu, and colleagues reported N‐doped CDs with yellow‐green solid‐state fluorescence by taking polyvinyl alcohol (PVA) and ethylenediamine (EDA) as starting materials via hydrothermal reaction.^[^ ^82^ ^]^ The plentiful PVA chains existed on the CDs’ surface could prevent the *π*–*π* interactions of the carbon cores, thus resisting the AIQ of the CDs (Figure 12c). Yang's group reported full‐color solid‐state fluorescent CDs through a one‐pot microwave‐assisted means in the presence of EDA and maleic acid (Figure 12d).^[^ ^111^ ^]^ It was found that crosslinking polymer net structures were formed during the formation of CDs. The net structures could efficiently prevent the aggregation of emission centers; meanwhile, the presence of non‐conjugated sub‐fluorophores were conducive to circumvent AIQ effect in aggregation state.

It is worth noting that CDs with high QYs in solid state do not mean high performance in LEDs. Solid‐state fluorescent CDs sometimes possess poor dispersibility in solvents, resulting in bad emissive layer films with rough surface and bad performance of LED devices. Besides, the presence of abundant long chain passivating ligands on CDs surface is also adverse for achieving high performance LEDs due to their poor transporting property, which will cause imbalanced hole and electron injections during the photoelectric conversion process. This kind of CDs generally own excitation‐dependent fluorescence emission behavior due to the surface defects. Therefore, ideal solid‐state fluorescent CDs for LED application should possess both high QYs, good dispersibility, good film‐forming property and outstanding transporting property, and there is still a long way for realizing it.

### 5.2 RTP CDs

As for LED displays, EQE is a critical merit for judging the device performance. The equation is as follows:

(1) $$\eta_{\mathrm{EQE}}=\eta_{\mathrm{out}}\times \eta_{\mathrm{IQE}}=\eta_{\mathrm{out}}\times \gamma \times \;\varphi_{\mathrm{PL}}\;\times \;\varphi_{\mathrm{exc}}$$

where *η*_out_ is light‐outcoupling efficiency, *ƞ*_IQE_ is internal quantum efficiency, *γ* is charge balance factor (*γ* = 1 in ideal condition), *φ*_PL_ is photoluminescence quantum yield, and *φ*_exc_ is efficiency of radiative exciton production (*φ*_exc_ = 25% in fluorescent materials and *φ*_exc_ = 100% in phosphorescent and TADF materials). In LED displays, *η*_out_ is ≈20% due to the surface plasmon losses and optical waveguide effect. As a consequence, the theoretically highest *ƞ*_EQE_ in fluorescent and phosphorescent CDs is of 5% and 20%, respectively. Therefore, employing phosphorescent CDs into LED devices is of great importance for practical application.

In the past several years, CDs with RTP property have attracted ever‐increasing interest and intense investigations concerning RTP CDs have been reported thereof. Generally, organic phosphorescence is related to the spin‐forbidden radiative transition from the lowest triplet state (T_1_) to ground state (S_0_).^[^ ^112^ ^]^ To endow CDs with phosphorescence, efficient intersystem crossing (ISC) from the lowest singlet state (S_1_) to T_1_ is of great importance, which is crucial for the generation of triplet excitons. So far, many strategies have been developed to promote the ISC process, such as restricting CDs in solid‐state matrixes,^[^ ^19^ ^]^ doping heteroatoms,^[^ ^20^ ^]^ and designing new synthetic methods.^[^ ^21^, ^22^ ^]^ In the past few years, the lifetimes of RTP CDs have achieved great progress from several hundreds of microseconds to a few seconds at room temperature under ambient conditions, which can be directly observed by naked eye.

**Figure 13:**
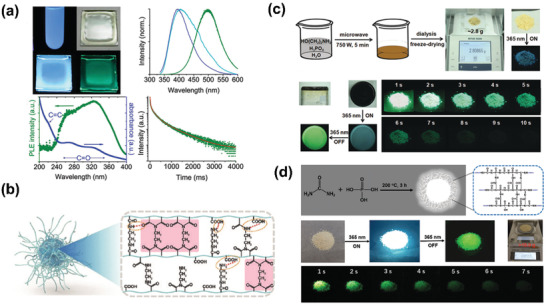
a) Digital photographs and the corresponding spectra of RTP CDs in PVA matrix. Reproduced with permission.^[^ ^19^ ^]^ Copyright 2013, Royal Society of Chemistry. b) Schematic illustration of the crosslink‐enhanced emission effect contributed to the formation of RTP CDs. Reproduced with permission.^[^ ^22^ ^]^ Copyright 2017, Wiley‐VCH. c) Schematic illustration of the preparation process for RTP CDs by microwave irradiation of ethanolamine, phosphoric acid and water. Reproduced with permission.^[^ ^20^ ^]^ Copyright 2018, Wiley‐VCH. d) Schematic diagram showing the preparation of fluorescence–phosphorescence dual emissive CDs via one‐step heat treatment process of urea and phosphoric acid aqueous solutions. Reproduced with permission.^[^ ^125^ ^]^ Copyright 2020, Wiley‐VCH.

The first RTP CDs was reported in 2013 by Zhao and colleagues.^[^ ^19^ ^]^ They realized RTP property of CDs by dispersing them into PVA matrix. The obtained CDs‐PVA composite possessed obvious phosphorescent emission at 500 nm under UV light with an average lifetime of 380 ms (**Figure** 13a). The phosphorescence was proposed to originate from the aromatic carbonyls of CDs, and the PVA matrix could effectively protect their triplet states from being quenched by hydrogen bond interaction. Science then, a variety of RTP CDs have been reported by blending CDs into host materials, such as PVA,^[^ ^113^, ^114^ ^]^ polyurethane,^[^ ^115^, ^116^ ^]^ potassium aluminum sulfate,^[^ ^117^ ^]^ recrystallized urea/biuret,^[^ ^118^ ^]^ and others.^[^ ^119^, ^120^, ^121^ ^]^ However, the blending strategy to achieve RTP is severely limited in practical applications due to the instability and compatibility problems between CDs and hosts in complex conditions. Besides, the poor conductivity and low thermal stability of host materials are not conducive to the preparation of LED devices. Hence, in consideration of the demands in practical applications, exploiting self‐protective RTP CDs is of great significance.

Taking advantage of crosslink‐enhanced emission effect,^[^ ^122^ ^]^ Yang and colleagues prepared a series of RTP CDs without the need of additional matrix complexation through hydrothermal treatment of polyacrylic acid and EDA (Figure 13b).^[^ ^22^ ^]^ The formed covalent crosslinking network could not only produce luminescence centers but also restrain their rotation and vibration, thus offering advantageous conditions for valid ISC. Besides, Lin and coworkers reported a microwave‐assisted heating method to facilely prepare RTP CDs in gram‐scale (Figure 13c).^[^ ^20^ ^]^ In this case, ethanolamine and phosphoric acid were used as precursors. The prepared CDs showed an ultralong RTP lifetime of 1.46 s. Later, the same group proposed a self‐immobilization strategy to transfer fluorescent CDs to RTP CDs through a simple heating process.^[^ ^123^ ^]^ They found the intraparticle hydrogen bonds could self‐immobilize excited triplet states and further promote the ISC process. Recently, Zhu et al. utilized seed growth method to achieve color‐tunable RTP CDs with emission wavelength in the range of 500–600 nm. The emission color ranged from blue, green, yellow to orange with the increase of CD sizes.^[^ ^124^ ^]^ It demonstrated the interparticle hydrogen bonds formed within the nitrogen‐containing groups on the surface of CDs could efficiently restrain the quenching of excited triplet states. Very recently, Fan's group reported single‐component white‐color carbon nitride quantum dots (W‐CNQDs) using urea as starting material.^[^ ^21^ ^]^ The as‐prepared W‐CNQDs exhibited blue‐yellow fluorescence–phosphorescence dual emissions with a QY of 25%. Detailed experimental characterizations together with theoretical calculations demonstrated that the presence of carbonyl groups at the edge of W‐CNQDs is crucial for inducing intermolecular electronic coupling and facilitating ISC process, thereby resulting in the yellow phosphorescence. In addition, Wang et al. also realized single‐component white‐color carbonized polymer dots (SW‐CPDs) by taking advantage of blue‐green fluorescence–phosphorescence dual emissions (Figure 13d).^[^ ^125^ ^]^ It was verified that the white‐color emission was constituted of fluorescence and phosphorescence two components. In solid power, a high QY of ≈41% for the white‐color emission was observed, while the QY of the phosphorescence in this case was 23%. However, to date, the reported RTP CDs mostly exhibit blue and green photoluminescence emissions. As we know, CDs with multicolor and long wavelength emission are of great necessity and significance in optoelectronic applications.

Though great achievements have been achieved concerning RTP CDs in the past several years, the applications of RTP CDs are mainly restricted in information encryption. To the best of our knowledge, employing RTP CDs as emissive layer in LED devices has not been reported so far. Besides, it should be point out that in LEDs, RTP CDs with short lifetimes are more advantageous for realizing high performance devices than long lifetime RTP CDs, due to the triplet–polaron or triplet–triplet exciton annihilation effects.

### 5.3 TADF CDs

TADF materials are regarded as the third generation‐luminescence materials, which possess high radiative decay rates and fluorescence efficiency by efficiently harvesting triplet states through reverse intersystem crossing (RISC) process from triplet (T1) to singlet (S1) states. Theoretically, the IQE of TADF materials can reach 100%. Therefore, TADF materials can serve as ideal emissive layer to construct high performance LED devices.

Since the first report of the application of TADF materials in OLEDs by Adachi et al. in 2012,^[^ ^126^ ^]^ rapid development and huge progress have been achieved in the past years.^[^ ^127^ ^]^ So far, TADF‐based electroluminescence LEDs with emission light covering the whole visible ranges have already been realized. Especially, the EQE of blue‐color and green‐color OLEDs has already exceeded 35%,^[^ ^128^, ^129^ ^]^ while nearly 30% in red‐color OLEDs.^[^ ^130^ ^]^ Meanwhile, the mechanism and design strategy of TADF also have been widely investigated, and numerous TADF substances including small organic molecules and polymers have been achieved thereof.

Despite the great achievements on TADF materials and TADF‐based OLEDs, the research on CDs‐based TADF materials is just at the infant stage that is full of challenges and opportunities. Tetsuka and coworkers first observed the delayed fluorescence phenomenon in amino‐functionalized GQDs. They found that resonance took place between the delocalized *π* orbital and molecular orbital in the NH_2_ group, which could effectively enhance the spin–orbit coupling and lead to ISC process.^[^ ^131^ ^]^ Later, Ghosh's group used liquid exfoliation strategy to achieve GQDs exhibiting both excitation‐independent, upconversion and delayed fluorescence property.^[^ ^132^ ^]^ The as‐prepared GQDs possessed a delay lifetime of 10 ± 2 µs in dimethyl formamide and water.

**Figure 14:**
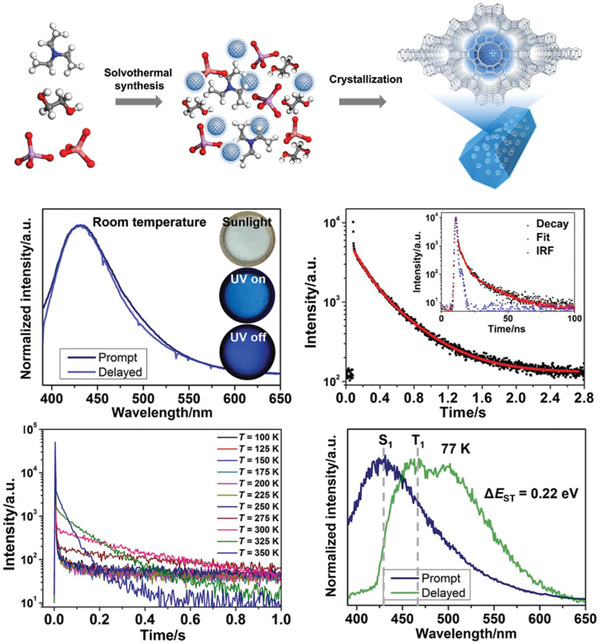
Schematic illustration of the formation process of CDs@zeolite composites and their TADF properties. Reproduced with permission.^[^ ^25^ ^]^ Copyright 2017, American Association for the Advancement of Science.

Using microwave heating method, Ding and coworkers prepared CDs with TADF performance with acetic acid as precursor in an immiscible system.^[^ ^24^ ^]^ The obtained CDs exhibited a blue emission at around 445 nm and excitation‐independent fluorescence property. At low temperature, CDs showing TADF property with a slow decay lifetime of 676–810 ns was observed. The cause of the TADF emission was due to the frozen solution, which could serve as solid matrix to efficiently restrain the nonradiative relaxation of CDs. Besides, with the temperature increasing from 100 to 250 K, the ratio of delayed constituent increased monotonically, further confirming the TADF behavior. Later, Liu et al. prepared TADF CD‐based hybrid materials by a dots‐in‐zeolites strategy (**Figure** 14).^[^ ^25^ ^]^ The TADF materials were fabricated by embedding CDs into zeolitic crystalline, with a high quantum yield of 52.14%. Interesting, the as‐prepared CDs@zeolite composites possessed long lifetimes up to 350 ms even at ambient conditions. Very recently, the same group achieved high‐efficient dual‐emission CDs@zeolite composites through an in‐situ solvent‐free thermal crystallization method.^[^ ^133^ ^]^ The obtained hybrids showed both ultralong RTP and TADF emission, with a lifetime up to 1.7 and 2.1 s, respectively. In addition, Lin and coworkers reported m‐CDs@nSiO2 composites with room temperature long afterglow of 0.703s through covalently bonding CDs and nanosilica (nSiO_2_).^[^ ^26^ ^]^ They demonstrated the formed covalent bonds could remarkably decrease the energy gap of CDs, thus leading to the TADF emission. He et al. used electrospinning technology to successfully prepare CD‐based composite films with both RTP and TADF property.^[^ ^134^ ^]^ SEM images demonstrated ordered mesoporous structure was formed in the obtained nanofibers during the electrospinning process. Further characterizations confirmed that the mesoporous structure could effectively stabilize the CDs’ triplet state and enable the RISC process. So far, only few references concerning CDs with TADF property have been reported. However, by learning from the valuable experience of TADF‐based OLEDs, we are convinced that various TADF CDs and their corresponding LED devices will be realized in the next future.

### 5.4 Interface Optimization Strategy

Nowadays, the main focus on CDs‐based LEDs is to improve the emissive layer quality, but the optimization of device structure has not been paid enough attention. In addition to the fabrication of high‐performance CDs‐based emissive layer, interface optimization of the photoelectric device is also important for achieving high‐quality LEDs, as already demonstrated in OLED, QLED, and PeLED areas. The carrier mobility and energy level structure of the interface transport layer directly determine the efficiency and balance of the carrier injection and the blocking effect of the reverse carrier. The selection of appropriate interface transport layer materials can ensure the formation of a better energy level gradient among emissive layer and transport layers, thus improving the performance of the device.

Park and coworkers prepared CDs‐based LEDs by using a simple device structure without any electron transporting and hole blocking layers.^[^ ^86^ ^]^ Though LEDs could be lighted in this case, the obtained devices were in very poor performance with maximum luminance of only 1 cd m^−2^. By introducing TFB as hole transport layer, Yuan et al. achieved high color‐purity deep‐blue LEDs with EQE_max_ of 4% and maximum luminance of 5240 cd m^−2^, which is the best performance among the reported blue‐color CD‐based electroluminescent LEDs.^[^ ^95^ ^]^ They found the introduction of TFB layer could remarkably improve the charge injection and balance in the active emission layer and thereby improve the device performance. Without TFB, the device performance decreased seriously, with EQE_max_ of 1.7% and maximum luminance of 954 cd m^−2^. The above cases strongly confirm the importance of interface layer materials in LED application. There is no doubt that the importance of interface optimization will be increasingly emphasized as high‐performance CDs are developed to a certain extent.

## 6 Conclusions and Outlook

Since first reported in 2004, CDs have been regarded as promising fluorescent materials on account of their outstanding photoluminescence properties, high stability, low toxicity, and easy preparation. These unique features endow CDs with significant potentials in future flat‐panel displays and solid‐state lighting. In this review, we mainly summarize the advances of CD‐based electroluminescent LEDs in the past decade, in which CDs are employed as active emission layer and interface transport layer materials is discussed and highlighted. Specifically, in view of the current relatively poor device performance of CD‐based electroluminescent LEDs, feasible strategies for constructing high performance CD‐based LEDs from the perspective of high‐quality CDs’ preparation (solid‐state fluorescent CDs, RTP CDs, and TADF CDs) and device structure optimization are proposed.

Though great achievements have been made focusing on CDs in the past decade, the exploration of CD‐based electroluminescent LEDs is still at the infant state and many issues are needed special attention, which may influence their future practical applications. First, CDs used in the manufacture of electroluminescent LEDs must be of exceptionally high purity. Nowadays, the widely used purification method is silica column chromatography. However, this method will cause huge‐cost, high toxicity and environmental pollution due to usage of large amount of organic solvents, which is also not suitable for the practical applications. As we know, cost is always the primary consideration to determine the feasibility of practical application. Therefore, more effort needs to be made to exploit novel efficient separation strategy. Second, the most popular route for preparing CDs is solvothermal method to date, which is conducted under high temperature and high pressure and not suitable for mass synthesis of CDs. How to acquire high‐quality CDs with both large‐scale synthesis and easy purification is of significant importance and deserves more attention. Delightedly, some groups have developed novel methods for preparing scale‐up CDs through unique reaction systems^[^ ^135^ ^]^ or techniques,^[^ ^136^ ^]^ which can be referred in future commercialization. Third, the detail photoelectric conversion mechanism in CD‐based LED devices and the exact structures and luminescent mechanisms of CDs are still unclear, which make it difficult to optimize the device architectures and obtain high‐quality electroluminescent LEDs. In summary, there is still a long way to go for future large‐scale commercialization of CD‐based electroluminescent LEDs. However, with the development of 5G network and artificial intelligence, increasing attention on novel fluorescent materials and next‐generation LED technology will be paid. Therefore, we firmly believe these challenges can be solved in near future and the progress of CDs and their corresponding LEDs will undoubtedly be enhanced. In the past decade, the foundation of CD‐based electroluminescent LEDs has been laid, and we highly expect their explosive development in the next decade.

## Conflict of Interest

The authors declare no conflict of interest.
